# Dynamically simulating the interaction of midazolam and the CYP3A4 inhibitor itraconazole using individual coupled whole-body physiologically-based pharmacokinetic (WB-PBPK) models

**DOI:** 10.1186/1742-4682-4-13

**Published:** 2007-03-26

**Authors:** Michaela Vossen, Michael Sevestre, Christoph Niederalt, In-Jin Jang, Stefan Willmann, Andrea N Edginton

**Affiliations:** 1 Competence Center Systems Biology, Bayer Technology Services GmbH, 51368 Leverkusen, Germany; 2 Competence Center Computational Solutions, Bayer Technology Services GmbH, 51368 Leverkusen, Germany; 3 Department of Pharmacology and Clinical Pharmacology Unit, Seoul National University College of Medicine and Hospital, Seoul, South Korea

## Abstract

**Background:**

Drug-drug interactions resulting from the inhibition of an enzymatic process can have serious implications for clinical drug therapy. Quantification of the drugs internal exposure increase upon administration with an inhibitor requires understanding to avoid the drug reaching toxic thresholds. In this study, we aim to predict the effect of the CYP3A4 inhibitors, itraconazole (ITZ) and its primary metabolite, hydroxyitraconazole (OH-ITZ) on the pharmacokinetics of the anesthetic, midazolam (MDZ) and its metabolites, 1' hydroxymidazolam (1OH-MDZ) and 1' hydroxymidazolam glucuronide (1OH-MDZ-Glu) using mechanistic whole body physiologically-based pharmacokinetic simulation models. The model is build on MDZ, 1OH-MDZ and 1OH-MDZ-Glu plasma concentration time data experimentally determined in 19 CYP3A5 genotyped adult male individuals, who received MDZ intravenously in a basal state. The model is then used to predict MDZ, 1OH-MDZ and 1OH-MDZ-Glu concentrations in an CYP3A-inhibited state following ITZ administration.

**Results:**

For the basal state model, three linked WB-PBPK models (MDZ, 1OH-MDZ, 1OH-MDZ-Glu) for each individual were elimination optimized that resulted in MDZ and metabolite plasma concentration time curves that matched individual observed clinical data. In vivo K_m _and V_max _optimized values for MDZ hydroxylation were similar to literature based in vitro measures. With the addition of the ITZ/OH-ITZ model to each individual coupled MDZ + metabolite model, the plasma concentration time curves were predicted to greatly increase the exposure of MDZ as well as to both increase exposure and significantly alter the plasma concentration time curves of the MDZ metabolites in comparison to the basal state curves. As compared to the observed clinical data, the inhibited state curves were generally well described although the simulated concentrations tended to exceed the experimental data between approximately 6 to 12 hours following MDZ administration. This deviations appeared to be greater in the CYP3A5 *1/*1 and CYP3A5 *1/*3 group than in the CYP3A5 *3/*3 group and was potentially the result of assuming that ITZ/OH-ITZ inhibits both CYP3A4 and CYP3A5, whereas in vitro inhibition is due to CYP3A4.

**Conclusion:**

This study represents the first attempt to dynamically simulate metabolic enzymatic drug-drug interactions via coupled WB-PBPK models. The workflow described herein, basal state optimization followed by inhibition prediction, is novel and will provide a basis for the development of other inhibitor models that can be used to guide, interpret, and potentially replace clinical drug-drug interaction trials.

## Background

Drug-drug interactions resulting from the inhibition of an enzymatic process can have serious implications for clinical therapy. Quantifying the magnitude of the inhibitor effect in vivo is an active area of study although methods of quantifying the exposure increase of a drug concomitantly administered with an inhibitor have focused on, until now, simplistic, static models [1-3]. These approaches assume that there is a proportional increase in exposure at high inhibitor concentrations and do not account for the time course of inhibitor concentrations. The approach that is taken is one which dynamically links inhibitor and drug models using whole-body physiologically-based pharmacokinetic models (WB-PBPK) to quantify, under any administration time and dose regimen, the changes that occur in parent compound exposure as well as the dynamic changes in the respective metabolite exposures. This has been done for midazolam (MDZ), and its two major metabolites 1' hydroxymidazolam (1OH-MDZ) and the glucuronide of 1' hydroxymidazolam (1OH-MDZ-Glu), in the presence of the CYP3A4 inhibitors itraconazole (ITZ) and its major metabolite hydroxy-itraconazole (OH-ITZ). This example was used because of the importance of CYP3A4 to drug metabolism and the availability of a full clinical data set for MDZ given in the basal and ITZ/OH-ITZ inhibited state [4].

Cytochrome P450 (P450) enzymes play an important role in the metabolism of exogenous and endogenous molecules. In humans, CYP3A represents one of the most important subfamilies of the P450 superfamily. CYP3A4 is the major P450 in the liver and intestine and has been reported to be involved in the metabolism of more than 60% of all medically relevant drugs [5]. The expression of CYP3A5 is highly polymorphic, due to a single nucleotide polymorphism, which is designated CYP3A5*3 [6]. Population frequencies for CYP3A5 variants in mixed American and Korean individuals are 61–77% for CYP3A5*3/*3, 22–33% for CYP3A5*1/*3 and 1–5% for CYP3A5*1/*1 [4,7] with CYP3A5*3/*3, CYP3A5*1/*3 and CYP3A5*1/*1 constituting 5%, 50% and 76% of the total CYP3A concentration, respectively. Total CYP3A content was more than 2-fold higher for livers with at least one CYP3A5*1 allele compared with CYP3A5*3/*3 livers [7]. Because CYP3A5 exhibits an overlapping substrate specificity with that of CYP3A4, it may contribute significantly to the metabolic elimination of CYP3A substrates in people carrying the wild-type CYP3A5*1 allele, although in vivo data as well as in vitro evidence are conflicting [4,8]. Because CYP3A is significantly involved in drug biotransformation, drug-drug interactions resulting from the inhibition of CYP3A-mediated metabolism by a co-administered therapeutic agent are of clinical importance.

**Figure 1:**
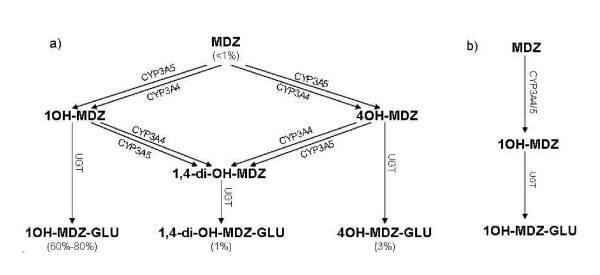
a) Schematic representation of the CYP3A4-, CYP3A5- and UGT-catalyzed metabolic pathway for MDZ. The percentages of the administered MDZ dose which are excreted in the urine within 24 hours [11,12] are given below the compounds. Abbreviations: MDZ, midazolam; 1OH-MDZ, 1-hydroxymidazolam; 4OH-MDZ, 4-hydroxymidazolam; 1,4-di-OH-MDZ, 1,4-dihydroxymidazolam; 1OH-MDZ-Glu, 1-hydroxymidazolam glucuronide; 1,4-di-OH-MDZ-Glu, 1,4-dihydroxymidazolam glucuronide; 4-OH-MDZ-Glu, 4-hydroxymidazolam glucuronide; UGT, uridine diphosphate-glucuronosyl-transferase. b) Schematic representation of the simplified metabolic pathway for MDZ used in the coupled model approach.

MDZ is a short-acting benzodiazepine that is primarily metabolized in the liver and gut wall by CYP3A4 and CYP3A5 [9,10]. The major active metabolite 1-hydroxymidazolam (1-OH-MDZ) and the minor metabolite 4-hydroxymidazolam (4-OH-MDZ) can be further hydroxylated to yield 1,4-dihydroxymidazolam (1,4-di-OH-MDZ) [9]. All metabolites are rapidly converted to their glucuronide conjugates by uridine diphosphate-glucuronosyl-transferases (UGTs) (Figure 1a) and excreted into the urine [10,11]. Within 24 h, 60% to 80% of a MDZ dose is excreted in the urine as 1-OH-MDZ-Glu, 3% as 4-OH-MDZ-Glu and 1% as 1,4-di-OH-MDZ-Glu [11,12]. No significant amounts of parent drug or primary metabolites are extractable from urine [12].

Itraconazole (ITZ) is an orally active triazole antimycotic agent, which is active against a broad spectrum of fungal species. ITZ is extensively metabolized in humans, yielding over 30 metabolites, including its primary active metabolite hydroxy-itraconazole (OH-ITZ). ITZ and its subsequent sequential metabolites [OH-ITZ, keto-itraconazole (keto-ITZ) and N-desalkyl-itraconazole (ND-ITZ)] are all high affinity ligands and substrates of CYP3A4 [13]. ITZ and OH-ITZ are also competitive inhibitors of CYP3A4. Keto-ITZ and ND-ITZ may also contribute to CYP3A4 inhibition in vivo following ITZ therapy, but their concentration following ITZ administration is significantly lower resulting in a low inhibitory influence [13].

In this study, we aim to predict the effect of ITZ and OH-ITZ CYP3A4 inhibition following oral ITZ administration on the pharmacokinetics of intravenously administered MDZ using a mechanistic WB-PBPK simulation model. WB-PBPK modeling allows for the simulation of the fate of xenobiotics in the human body on the basis of individual physiological characteristics [14]. The model is build on MDZ, 1OH-MDZ and 1OH-MDZ-Glu plasma concentration time data experimentally determined in 19 CYP3A5 genotyped adult male individuals, who received MDZ intravenously in basal and ITZ-inhibited CYP3A metabolic states [4]. At first, three WB-PBPK models, one for MDZ, one for 1OH-MDZ and one for 1OH-MDZ-Glu, will be coupled per individual to dynamically simulate the kinetics of MDZ hydroxylation and glucuronidation for each study volunteer. In a second step, the MDZ models will be extended by linking WB-PBPK models of ITZ and OH-ITZ in order to predict the interaction between MDZ and ITZ via ITZ and OH-ITZ-mediated CYP3A4-inhibition in a time-dependent manner.

## Results

**Figure 2:**
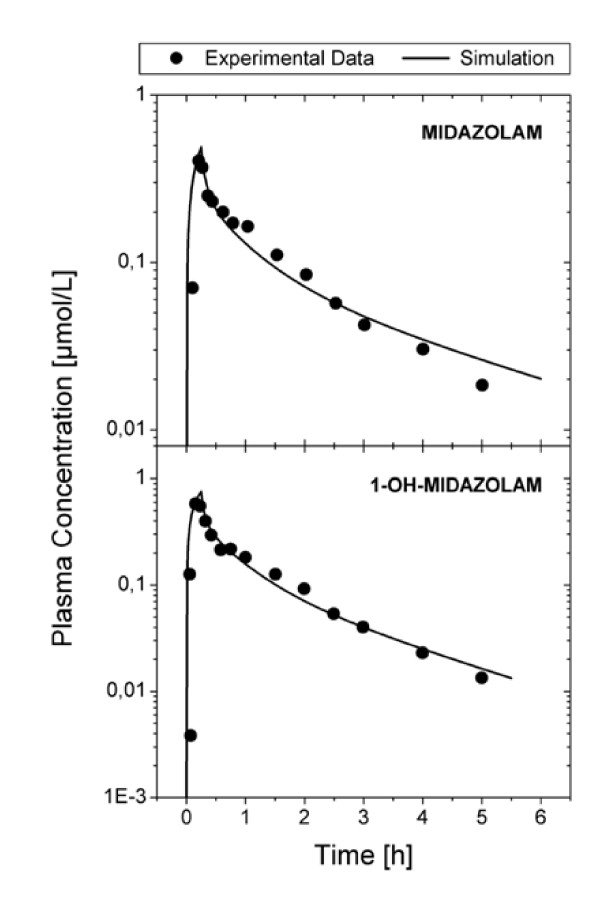
Simulated plasma concentration time curve (line) of a) MDZ following a 15 minute intravenous infusion of 0.1 mg/kg MDZ and b) 1OH-MDZ following a 15 minute intravenous infusion of 0.15 mg/kg 1OH-MDZ as compared to mean experimental data. Experimental data was taken from Mandema et al. [15].

The simulated plasma concentration time curves following intravenous administration of MDZ and 1OH-MDZ adequately represented the corresponding in vivo time course data reported by Mandema et al. [15] (Figure 2).

**Figure 3:**
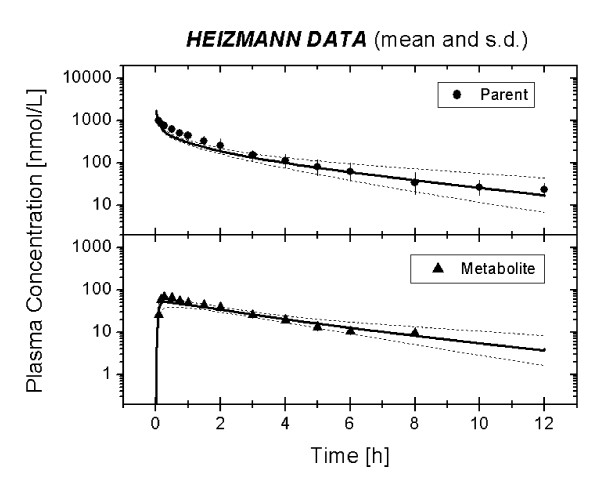
Mean of the predicted plasma concentration time curves (line) of MDZ and 1OH-MDZ following an intravenous administration of 0.15 mg/kg as compared to the mean (*n *= 6) of the experimental data. Experimental data was taken from Heizmann et al. [11].

Figure 3 presents the mean (± standard deviation) of the six individual optimized plasma concentration time profiles for MDZ and 1OH-MDZ (lines) along with the mean (± standard deviation) of the experimental data (symbols) [11]. The elimination optimized curves matched the experimental data well. Table 1 presents the minimum, mean and maximum velocity-rate constants (k_1,-1,2_), K_m_, V_max,_V_max_/K_m _and CL(1OH) values which were the results of the individual optimizations for the six individuals.

**Table 1:** Mean, minimum and maximum parameter values from the optimization of the coupled WB-PBPK model using the Heizmann et al. [11] data (*n *= 6) and the Kharasch et al. [16] mean data.

		Heizmann		Kharasch
	
Parameter	Mean	Min	Max	Mean
1OH_k_1 _[L·umol^-1^·min^-1^]	5.6E^-2^	4.1E^-2^	7.4E^-2^	2.7E^-2^
1OH_k_-1 _[min^-1^]	8.1E^-6^	4.3E^-21^	4.8E^-5^	1.1E^-11^
1OH_k_2 _[min^-1^]	0.12	0.086	0.24	0.14
CL(1OH) [L·min^-1^]	75.1	56.7	97.4	
1OH_K_m _[umol·L^-1^]	2.2	1.4	3.9	5.3
1OH_V_max _[umol·min^-1^·g_tissue_^-1^]	3.4E^-4^	2.2E^-4^	6.6E^-4^	4.0E^-4^
Glu_k_1 _[L·umol^-1^·min^-1^]				1.1
Glu_k_-1 _[min^-1^]				2.6E^-9^
Glu_k_2 _[min^-1^]				0.11
Glu_K_m _[umol·L^-1^]				0.10
Glu_V_max _[umol·min^-1^·g_tissue_^-1^]				7.7E^-5^
CL(Glu) [L·min^-1^]				14.5

**Figure 4:**
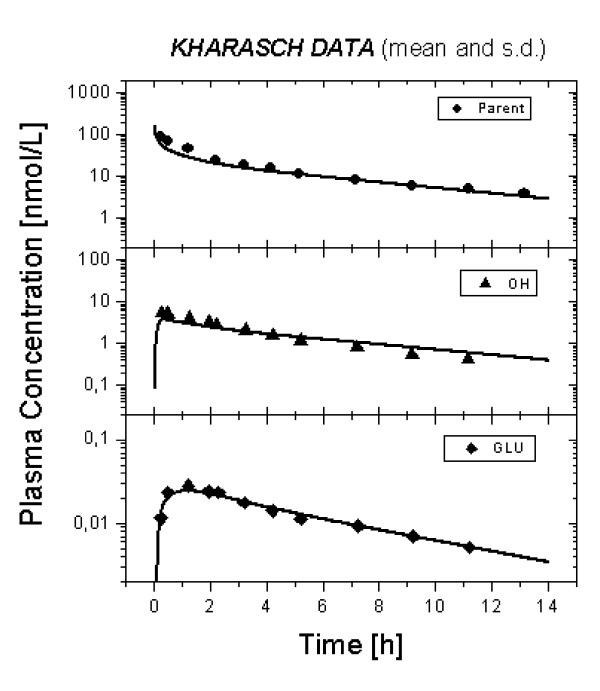
Predicted plasma concentration time curves (lines) of MDZ, 1OH-MDZ and 1OH-MDZ-Glu following an intravenous bolus administration of 1 mg MDZ as compared to experimental data from a typical individual (symbols). Experimental data was taken from Kharasch et al. [16].

The optimized plasma concentration time profiles for MDZ, 1OH-MDZ and 1OH-MDZ-Glu (lines) are presented in Figure 4 together with the experimental data (symbols) [16]. The elimination optimized curves well represented the experimental data. Numerical results are shown in Table 1.

**Figure 5:**
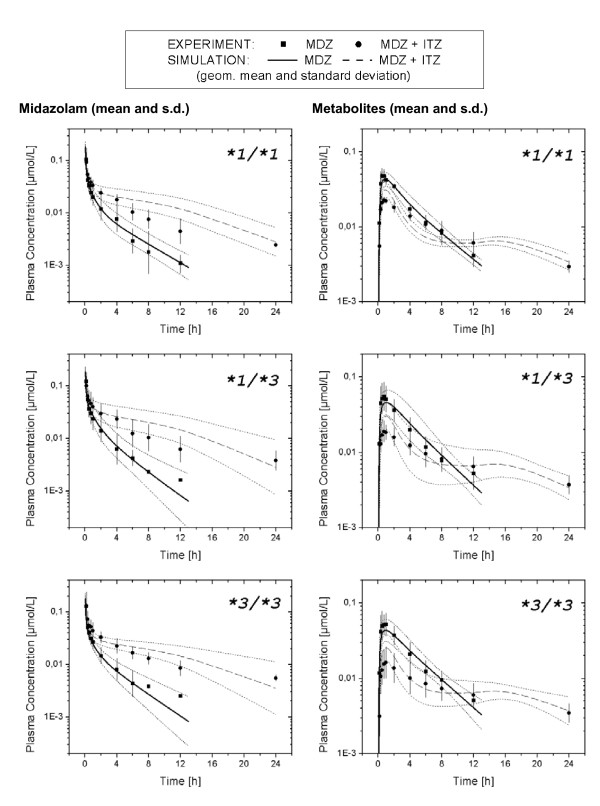
Mean ± standard deviation plots of the experimental observed plasma concentration time profiles for MDZ and the sum of its metabolites, 1OH-MDZ and 1OH-MDZ-Glu (symbols: squares) and the corresponding mean elimination optimized curves (solid lines) for the CYP3A5 genotypes, CYP3A5 *1/*1 (*n *= 6), CYP3A5 *1/*3 (*n *= 6) and CYP3A5*3/*3 (*n *= 7) in the basal state. Also presented is the mean ± standard deviation plots of the experimental observed plasma concentration time profiles for MDZ and the sum of its metabolites, 1OH-MDZ and 1OH-MDZ-Glu (symbols: circles) and the corresponding mean predicted curves (dotted lines) for the CYP3A5 genotypes in the CYP3A inhibited state resulting from ITZ administration. Plasma concentration time curves in the inhibited state were graphed starting at time = 0 to allow for direct comparison with the basal state curves. Experimental data was taken from Yu et al. [4].

Figure 5 presents the mean (± standard deviation) optimized plasma concentration time profiles for MDZ and the sum of 1OH-MDZ and 1OH-MDZ-Glu (lines) along with the mean experimental data (symbols) separated by the CYP3A5 genotype group [4]. The elimination optimized curves well represented the experimental data. The mean [range] of the resulting 1OH_K_m _and 1OH_V_max _values for the hydroxylation were 2.1 umol/L [0.69–5.0] and 3.4E^-4 ^umol/min [1.0E^-4 ^– 7.2E^-4^], respectively. For the glucuronidation, the mean [range] of the resulting Glu_K_m _and Glu_V_max _values were 0.21 umol/L [1.2E^-4 ^–1.5] and 4.0E^-3 ^umol/min [2.0E^-5^–4.7E^-2^], respectively. The mean [range] of the optimized renal elimination of 1OH-MDZ-Glu was 13.2 L/min [5.1–34.9].

**Figure 6:**
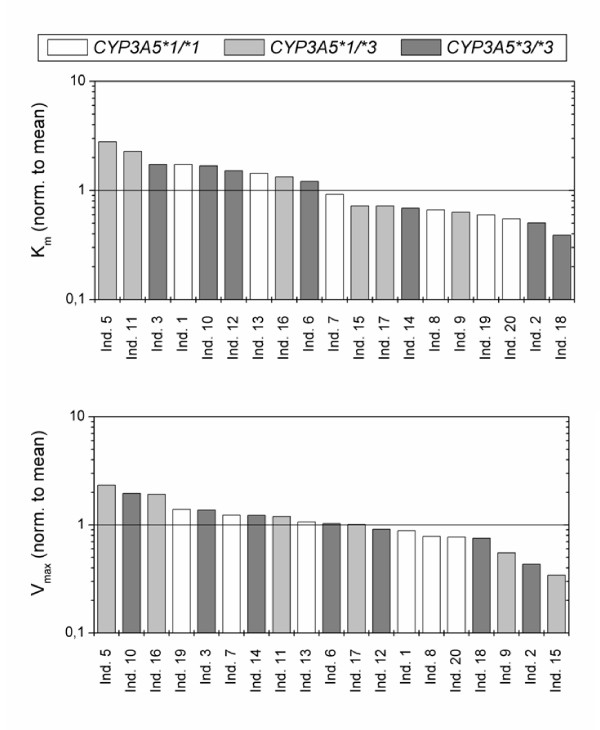
Rank-order plots for the optimized 1OH_K_m _and 1OH_V_max _values normalized to the mean.

In Figure 6, the individuals from the Yu et al [4] are ranked according to their K_m _and V_max _values for the hydroxylation, normalized to the mean. There is no correlation between the CYP3A5 genotype and the affinity of MDZ to CYP3A (K_m_) or the rate of hydroxylation (V_max_) in the basal state.

**Figure 7:**
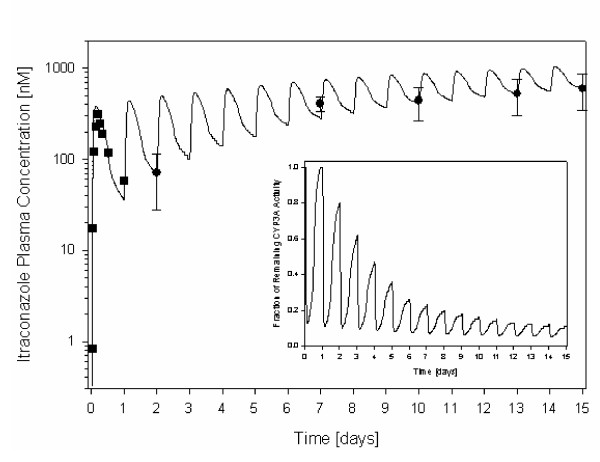
Simulated (line) ITZ plasma concentration time curve following 15 days of 200 mg once per day ITZ administration in the fed state. Mean experimental data (symbols: squares) for the first day was taken from Barone et al. [17] and experimental data (mean ± SD, symbols: circles) was taken from Hardin et al. [18]. The inset graph presents the estimated fraction of remaining CYP3A activity over time as a result of ITZ and OH-ITZ inhibition.

Figure 7 presents the simulated ITZ plasma concentration time curve following 200 mg dosing of ITZ in the fed state as compared to experimental data from Barone et al. [17] for the first day and Hardin et al. [18] for the trough concentrations on subsequent days. The fraction of remaining CYP3A activity resulting from the simulated unbound intracellular concentrations of ITZ and OH-ITZ in the liver over time is also presented in Figure 7.

Figure 5 also presents the mean (± standard deviation) predicted plasma concentration time profiles for MDZ and the sum of 1OH-MDZ and 1OH-MDZ-Glu (lines) along with the mean experimental data (symbols) separated by the CYP3A5 genotype group following ITZ administration [4]. MDZ exposure increases with a consequent decrease in initial 1OH-MDZ+1OH-MDZ-Glu concentrations and increase at later time points. While the shape of the curve is generally well described, the simulated concentrations tend to exceed the experimental data between approximately 6 to 12 hours. This deviations appears to be greater in the CYP3A5 *1/*1 and CYP3A5*1/*3 group than in the CYP3A5 *3/*3 group.

## Discussion

A dynamic, coupled WB-PBPK model of MDZ and its metabolites was developed to predict the time-dependent interaction of MDZ when co-administered with the CYP3A4 inhibitor ITZ. This novel method differs greatly from previously used models to identify the potential for drug-drug interactions. Static models generally prevail where competitive and irreversible inhibition is defined by the kinetic parameters of the inhibitor and are only dose dependent in relation to the inhibitor [2,3]. Using the static model, a ratio of basal state area under the curve (AUC) to inhibited state AUC is calculated, which provides a means of estimating the mean extent of inhibition in a given time period. Identifying parent compound and metabolite curve shape changes along with the generation of any metabolite profiles is not possible using this method. Since the static nature of these models limits their flexibility, dynamically interacting WB-PBPK models were seen as the method of choice for understanding how drug-drug interactions affect pharmacokinetic profiles of both the parent compound and its metabolites. Recently, Özdemir et al. [19] presented an attempt to predict the drug-drug interaction of MDZ with another antifungal agent, ketoconazole by means of the AUC increase of MDZ when co-administered with KTZ, and concluded that comprehensive PBPK models are required to correctly predict the inhibitory effect of ketoconazole.

The study of Yu et al. [4] provided an excellent data set of anthropometric (weight, height, age) data, CYP3A5 genotype and plasma concentration profiles for 19 individuals given MDZ alone and in combination with ITZ. Using this experimental data, each basal state individual MDZ and metabolite profile was optimized and, followed by the addition of the ITZ data and models, was used to test the model's ability to predict the MDZ-ITZ drug-drug interaction potential.

In a first step, the MDZ plus metabolite model was developed. Distribution properties of MDZ, 1OH-MDZ and 1OH-MDZ-Glu in this study were based on previously established mechanistic models to estimate organ partitioning parameters [14,20,21]. Four out of 17 organ partition coefficients were ultimately modified using experimental partition coefficients for MDZ scaled from rats [22]. For MDZ and 1OH-MDZ, this modification accurately led to simulated plasma concentration time profiles that mirrored observed profiles following IV administration.

The subsequent coupling of MDZ, 1OH-MDZ and 1OH-MDZ-Glu was achieved using velocity rate constants for metabolism parameterization. Using a step wise process inclusive of different experimental data sets [11,16], the velocity rate constants that defined the basal state kinetics of MDZ, 1OH-MDZ and 1OH-MDZ-Glu, were optimized for our study data [4]. In order to evaluate the optimization results, 1OH_K_m _values were compared to in vitro findings. The mean ± SD 1OH_K_m _values for the Heizmann et al. [11], Kharasch et al. [16] and Yu et al. [4] data sets were 2.2 ± 0.99 umol/L, 5.3 umol/L and 2.1 ± 1.17 umol/L, respectively. These were all similar to each other and also to the in vitro mean ± SD 1OH_K_m _= 3.9 ± 3.1 umol/L reported by Patki et al. [23]. The coefficient of variability (CV%) for 1OH_K_m _and 1OH_V_max _were also compared to in vitro findings to evaluate the reasonability of the generated inter-individual variation. The in vitro CV% for 1OH_K_m _and 1OH_V_max _generated from human liver microsomes (HLMs) of twelve individuals being 80% and 58%, respectively [23], were similar to the results of the optimization of the Yu et al. [4] data, namely 1OH_K_m _CV% = 57% and 1OH_V_max _CV% = 47%. Therefore, there is a correlation between in vitro and in vivo 1OH_K_m _and 1OH_V_max _for the hydroxylation of MDZ via CYP3A4/5. Furthermore, the individual 1OH_V_max _variability was linked to the underlying process by comparing the CV% of 1OH_V_max _with that of in vitro hepatic CYP3A4/5 concentration. The CV% for the CYP3A4/5 concentration in a group of Japanese, Caucasian and mixed Americans was 160% [23,24] whereas that for the Japanese population alone was 35% [24]. The CV% of V_max _for the Koreans of the Yu et al. [4] data, 47%, is between both values and closer to that for the Japanese population. This suggests that our model was able to capture the inter-individual variability in hepatic CYP3A concentration using 1OH_V_max _as a surrogate.

The enzyme responsible for the glucuronidation of 1OH-MDZ is unknown. In order to estimate the relevant UDP-glucuronosyltransferase (UGT) concentration, the findings by Reinach et al. [25] were used. They observed that the metabolism of MDZ was 0.080 nmol/min/10^6 ^cells while the metabolism of 1OH-MDZ was 0.020 nmol/min/10^6 ^cells in human hepatocytes. As there was no additional information available, for our study, it was assumed that the difference between the metabolism of MDZ and 1OH-MDZ was only dependent on differing enzyme concentration. Under this assumption, the UGT concentration was estimated, namely [UGT] = 0.25 [CYP3A]. The absolute Glu_K_m _and Glu_V_max _values are therefore provisional. Nevertheless, the optimized curves will not change when the actual UGT concentration is known, as the product of the velocity-rate constants and the enzyme concentration were optimized. Thus, if the enzyme concentration changes, then k_1_, k_-1 _and k_2 _can change accordingly (see equation 1) therefore changing the calculated K_m _(see equation 2, no steady state assumption is made) and V_max _values (see equation 3). Despite that the absolute K_m _and V_max _values are dependent on the enzyme concentration, inter-individual variability is independent of the absolute values. It is remarkable that the CV% of Glu_K_m _was relative high with 191%. For Glu_V_max_, the CV% equaled 299%. This was substantially higher than the CV% for 1OH_V_max_. As the CV% for V_max _can be linked to the underlying process, or more precisely the enzyme concentration, it can be inferred that the inter-individual variability of the corresponding UGT concentration might be higher than that of the CYP3A concentration. This hypothesis cannot be confirmed nor rejected because the UGT(s) catalyzing the glucuronidation of 1OH-MDZ or similar compounds are unknown. Once the UGT(s) catalyzing the glucuronidation of 1OH-MDZ and its (their) concentration is known, Glu_K_m _and Glu_V_max _can be recalculated and further interpreted.

The information about the contribution of CYP3A5 to overall MDZ metabolism is controversial. According to Huang et al. [8], CYP3A5 accounts for 27% of the total product formation catalyzed by microsomes with at least one CYP3A5*1 allele, whereas Williams et al. [26] reports that CYP3A4 and CYP3A5 contribute equally to the MDZ elimination. Other in vitro studies demonstrate that CYP3A5 has a higher catalytic activity but a lower affinity than CYP3A4 for MDZ 1-hydroxylation [8,23,26]. Despite the fact that the in vitro studies consistently suggested an influence of the CYP3A5 concentration on the MDZ elimination, in vivo findings have been inconsistent. A higher MDZ elimination for white cancer patients [27] and healthy Asian subjects [28] having at least one wild-type CYP3A5*1 allele compared to CYP3A5*3/*3 patients have been reported. In contrast, Floyd et al. [29] and Shih & Huang [30] found no effect of the CYP3A5 genotype on the elimination of MDZ in a mixed population of healthy white and African American adults and Chinese volunteers, respectively. Figure 6 represents the rank-order plot for the optimized 1OH_K_m _and 1OH_V_max _values for the Yu et al. [4] data. The result of this figure supports the hypothesis that CYP3A5 plays little to no role in MDZ hydroxylation and is the same result presented in the Yu et al. [4] study in the basal state.

In a second step, a WB-PBPK model for ITZ and OH-ITZ was developed and linked to in vitro data of their CYP3A4 inhibition potential. Through dynamically linking the inhibitor models with MDZ, and thus the MDZ-metabolite models, a prediction of the effect of ITZ given at 200 mg per day over 5 days could be made for each individual from the Yu et al. [4] study. One assumption of our model was that ITZ inhibits total CYP3A activity whereas it is actually specific to the inhibition of CYP3A4. The potential consequences of this were observed when MDZ was modeled in the presence of ITZ and OH-ITZ. There was a slight overprediction of the mean predicted plasma concentrations in relation to those observed between 6 and 12 hours, which was greater in individuals with at least one CYP3A5*1 allele. Since these individuals have a greater proportion of CYP3A5 relative to their total CYP3A content, this suggests that CYP3A5 remains un-inhibited by ITZ and OH-ITZ and thus can contribute to MDZ elimination. By comparing the observed and predicted MDZ pharmacokinetic profiles in the basal and inhibited states, the relative contribution of CYP3A5 to overall MDZ elimination in vivo is low regardless of the CYP3A5 genotype. Even in light of this model simplification, the resulting simulations adequately predicted the changes in the MDZ profiles as well as predicting the completely different MDZ metabolite profiles in the inhibited state.

While dynamic simulations of drug-drug interactions are desirable, their conceptualization and implementation are complex compared to the static AUC ratio method, as described above. Major limitations are the availability of pharmacokinetic data for the compound of interest and the inhibitor (especially if multiple metabolites are of concern) as well as a lack of thoroughly studied in vitro inhibition data. Simplification of the model is necessary when this data is not available, which may limit the flexibility and application of the model. In our case, the simplification of the CYP3A complex is one such example. The data that was required to incorporate both a CYP3A4 and CYP3A5 enzyme into the liver was limited by a lack of data on how much each enzyme contributed to 1OH-MDZ plasma concentrations. Further, no individual ITZ plasma concentration time data was available and thus a mean curve had to be generated and used for each individual. Knowing these limitations ahead of gathering the experimental data, may help to inform the clinical drug-drug interaction trial by guiding the planning stage.

## Conclusion

This study represents the first attempt to dynamically simulate metabolic enzymatic drug-drug interactions via coupled WB-PBPK models. The workflow described herein, basal state optimization followed by inhibition prediction, is novel and will provide a basis for the development of other inhibitor models that can be used to guide, interpret, and potentially replace clinical drug-drug interaction trials.

## Methods

### WB-PBPK model development and parameterization for defining MDZ CYP3A-mediated metabolism

PK-Sim^® ^(ver 3.0, Bayer Technology Services GmbH, Leverkusen, Germany) was used to generate the individual WB-PBPK models for MDZ, 1OH-MDZ and 1OH-MDZ-Glu. PK-Sim^® ^is a commercially available software tool for WB-PBPK modeling of drugs in laboratory animals and humans. It uses validated physiological models to estimate substance-specific absorption [21] and distribution parameters, such as organ/plasma partition coefficients and permeability coefficients, from physico-chemical properties of a compound such as lipophilicity, plasma protein binding, molecular weight and solubility [14,20,21]. Physiological databases are included in the software that incorporate the dependencies of organ weights, organ blood flows and intestinal parameters [gastrointestinal length, radius of each section, intestinal surface area [21]] with the weight and height of the individual [31]. For a detailed description of the WB-PBPK model structure implemented in PK-Sim^®^, see Willmann et al. [14,20,21].

Individual coupled WB-PBPK models, inclusive of MDZ and its metabolites, were set up. First, the MDZ WB-PBPK model was parameterized by inputting the physico-chemical properties of MDZ (Table 2), the weight of the individual, a mean height and the application regime into PK-Sim^®^. The MDZ WB-PBPK model contains a hepatic elimination process, defined in the liver intracellular space, describing the CYP3A mediated elimination of MDZ to its metabolite, 1OH-MDZ. This metabolism process was defined in the model assuming Michaelis-Menten kinetics (Figure 1b). The Michaelis-Menten mechanism for enzyme catalysis takes into account that the substrate [S] and the enzyme [E] first form a complex [ES] before the substrate is converted into the product [P]:

**Table 2:** Physicochemical properties of MDZ, 1OH-MDZ, 1OH-MDZ-Glu and ITZ.

Parameter	MDZ	1OH-MDZ	1OH-MDZ-Glu	ITZ
Lipophilicity (Log MA)^a^	2.9	2.6	1.4	3.84
f_unbound_	0.024	0.03	0.03	0.016^b^
Molecular weight (g/mol)	325.8	341.8	517.9	705.6
Effective molecular weight (g/mol)	286.8 (1Cl, 1F)	302.8 (1Cl, 1F)	478.9 (1Cl, 1F)	661.6 (2Cl)

^a ^MA = Membrane Affinity

^b ^Experimental value taken from [42].

E0+S⇄k−1k1ES→k2E0+P
 MathType@MTEF@5@5@+=feaafiart1ev1aaatCvAUfKttLearuWrP9MDH5MBPbIqV92AaeXatLxBI9gBaebbnrfifHhDYfgasaacH8akY=wiFfYdH8Gipec8Eeeu0xXdbba9frFj0=OqFfea0dXdd9vqai=hGuQ8kuc9pgc9s8qqaq=dirpe0xb9q8qiLsFr0=vr0=vr0dc8meaabaqaciaacaGaaeqabaqabeGadaaakeaacqqGfbqrdaWgaaWcbaGaeGimaadabeaakiabgUcaRiabbofatnaao0aaleaacqqGRbWAdaWgaaadbaGaeGymaedabeaaaSqaaiabbUgaRnaaBaaameaacqGHsislcqaIXaqmaeqaaaGccaGLsgIaayjKHaGaeeyrauKaee4uam1aa4ajaSqaaiabbUgaRnaaBaaameaacqaIYaGmaeqaaaWcbeGccaGLsgcacqqGfbqrdaWgaaWcbaGaeGimaadabeaakiabgUcaRiabbcfaqbaa@4440@

The micro-constants k_1_, k_-1 _and k_2 _are velocity-rate constants for the association of substrate and enzyme, the dissociation of unaltered substrate from the enzyme and the dissociation of product from the enzyme, respectively. At steady-state the following equation is valid,

k_1 _[E_0_][S] = k_-1_[ES] + k_2_[ES]     (1)

with [I] being the concentration of the compound I. The Michaelis-Menten constant K_m _can be calculated from the micro-constants as follows:

Km=k-1+k2k1.     (2)
 MathType@MTEF@5@5@+=feaafiart1ev1aaatCvAUfKttLearuWrP9MDH5MBPbIqV92AaeXatLxBI9gBaebbnrfifHhDYfgasaacH8akY=wiFfYdH8Gipec8Eeeu0xXdbba9frFj0=OqFfea0dXdd9vqai=hGuQ8kuc9pgc9s8qqaq=dirpe0xb9q8qiLsFr0=vr0=vr0dc8meaabaqaciaacaGaaeqabaqabeGadaaakeaacqqGlbWsdaWgaaWcbaGaeeyBa0gabeaakiabg2da9maalaaabaGaee4AaS2aaSbaaSqaaiabb2caTiabbgdaXaqabaGccqGHRaWkcqqGRbWAdaWgaaWcbaGaeeOmaidabeaaaOqaaiabbUgaRnaaBaaaleaacqqGXaqmaeqaaaaakiabc6caUiaaxMaacaWLjaWaaeWaaeaacqaIYaGmaiaawIcacaGLPaaaaaa@3E52@

The maximum velocity of the reaction (V_max_) depends on the total enzyme concentration [E]_total _and the rate of the second reaction step:

V_max _= k_2_[E]_total _    (3)

The intrinsic clearance (CL) is the rate constant of metabolization and is defined by the Michaelis-Menten equation:

CL=Vmax⁡[S]Km+[S]     (4)
 MathType@MTEF@5@5@+=feaafiart1ev1aaatCvAUfKttLearuWrP9MDH5MBPbIqV92AaeXatLxBI9gBaebbnrfifHhDYfgasaacH8akY=wiFfYdH8Gipec8Eeeu0xXdbba9frFj0=OqFfea0dXdd9vqai=hGuQ8kuc9pgc9s8qqaq=dirpe0xb9q8qiLsFr0=vr0=vr0dc8meaabaqaciaacaGaaeqabaqabeGadaaakeaacqqGdbWqcqqGmbatcqGH9aqpdaWcaaqaaiabbAfawnaaBaaaleaacyGGTbqBcqGGHbqycqGG4baEaeqaaOGaei4waSLaee4uamLaeiyxa0fabaGaee4saS0aaSbaaSqaaiabb2gaTbqabaGccqGHRaWkcqGGBbWwcqqGtbWucqGGDbqxaaGaaCzcaiaaxMaadaqadaqaaiabisda0aGaayjkaiaawMcaaaaa@4430@

Under the condition that the substrate concentration [S] is much smaller than K_m_, the intrinsic clearance approaches the ratio of V_max _and K_m_:

CL=Vmax⁡Km,if [S] << Km     (5)
 MathType@MTEF@5@5@+=feaafiart1ev1aaatCvAUfKttLearuWrP9MDH5MBPbIqV92AaeXatLxBI9gBaebbnrfifHhDYfgasaacH8akY=wiFfYdH8Gipec8Eeeu0xXdbba9frFj0=OqFfea0dXdd9vqai=hGuQ8kuc9pgc9s8qqaq=dirpe0xb9q8qiLsFr0=vr0=vr0dc8meaabaqaciaacaGaaeqabaqabeGadaaakeaafaqabeqacaaabaGaee4qamKaeeitaWKaeyypa0ZaaSaaaeaacqqGwbGvliGbc2gaTjabcggaHjabcIha4bGcbaGaee4saS0aaSbaaSqaaiabb2gaTbqabaaaaOGaeeilaWcabaGaeeyAaKMaeeOzayMaeeiiaaIaee4waSLaee4uamLaeeyxa0LaeeiiaaIaeeipaWJaeeipaWJaeeiiaaIaee4saS0aaSbaaSqaaiabb2gaTbqabaaaaOGaaCzcaiaaxMaadaqadaqaaiabiwda1aGaayjkaiaawMcaaaaa@4A1F@

Furthermore, the simplified model includes a CYP3A complex, which represents both iso-enzymes (CYP3A4 and CYP3A5) in the human liver. It is not possible to model the reactions catalyzed by CYP3A4 and CYP3A5 separately, as it is not clearly understood how much each contributes to the production of 1OH-MDZ. Therefore, an assumption has to be made, namely that the CYP3A complex is sufficient to model MDZ-ITZ interactions.

Following generation of a MDZ WB-PBPK model, the WB-PBPK models for 1OH-MDZ and 1OH-MDZ-Glu were parameterized by inputting 1OH-MDZ and 1OH-MDZ-Glu physico-chemical data (Table 2). All physiological model parameters such as age, weight, height and all depending parameters such as organ weights and blood-flow rates remained the same as in the MDZ model of the same individual. Since 1OH-MDZ is cleared in the liver via glucuronidation, the 1OH-MDZ WB-PBPK model contains a hepatic elimination of 1OH-MDZ to its glucuronide, 1OH-MDZ-Glu via UGT, defined by velocity rate constants (k_1,-1,2_) as described above in Eq.(1). Elimination of 1OH-MDZ-Glu [CL(Glu)] from the circulation was defined by a first order intrinsic elimination process in the kidney. Coupling of the three WB-PBPK models was done such that the source function generating 1OH-MDZ in the liver intracellular volume was equal to the output of the CYP3A mediated elimination of MDZ (on a molar basis). In the same way, the source function of 1OH-MDZ-Glu was equal to the output of the UGT mediated elimination of 1OH-MDZ within the liver intracellular space.

### Enzyme concentration

Hepatic enzyme concentrations in the individuals from where the study data was derived were unknown. Thus, typical enzyme concentrations had to be taken from the literature. The median CYP3A complex concentration of a mixed and a Japanese population was used as the enzyme concentration for the hydroxylation step in this study, such that [E_0_] equaled 70 pmol/mg_microsomal protein _[7,23,24]. The value 40 mg protein/g_liver _[32-34] was used to yield [CYP3A] = 2.8 nmol/g_liver_. The specific enzyme responsible for the glucuronidation of 1OH-MDZ is not known. Reinach et al. [25] measured the metabolism of MDZ using thawed human hepatocytes and found that the metabolism of MDZ to 1OH-MDZ is four times higher than the metabolism of 1OH-MDZ to 1OH-MDZ-Glu. This ratio was used to estimate a UGT concentration, namely [UGT] = 0.25× [CYP3A]. These enzyme concentration values remained the same for all individuals. Therefore, all of the variability in calculated V_max _values stem from k_2 _(see equation 3). During the simulations, the available enzyme concentration [E_0_] dynamically changes depending on the substrate concentration although the condition that [E]_total _= [E_0_] + [ES] always holds true.

### Volume of distribution

The volume of distribution is an important parameter in pharmacokinetic studies. Distribution volumes in PK-Sim^® ^are estimated based on physico-chemical data (Table 2), as described in Willmann et al. [20]. Membrane affinity, as a lipophilicity value, and plasma unbound fraction were assessed for MDZ and 1OH-MDZ using the Nimbus Technology (Nimbus Biotechnology, Leipzig, Germany) [35,36]. 1OH-MDZ-Glu is not commercially available and there exists no published lipophilicity or unbound fraction in plasma value. Therefore, the lipophilicity of 1OH-MDZ-Glu was estimated by using the mean logarithmic difference of the lipophilicities of other hydroxylated compounds and their corresponding glucuronides that were taken from the literature [37] (Table 2). The glucuronide of valproic acid has an ex vivo unbound fraction in serum that is half that of the parent compound [38]. In another study that examined the protein binding of three compounds and their glucuronides, the unbound fraction of the glucuronide increased by factors of 2.2, 1.3 and 2.6 [39]. For the purposes of the present study, the fraction unbound in plasma for 1OH-MDZ-Glu was kept the same as that of 1OH-MDZ (Table 2).

**Figure 8:**
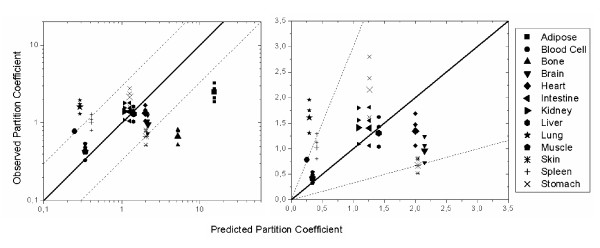
Comparison of observed and predicted tissue/plasma partition coefficients for the various organs (left: log-log plot, right: enlarged linear plot of the interval [0,3.5]). Small symbols indicate individual data reported by Björkmann et al. [22], large symbols denote the mean value for each organ. The solid line represents the identity, the dotted lines mark the region 3-fold-off the identity.

Plasma concentration time data was located for individuals who received either MDZ or 1OH-MDZ intravenously [15] and experimental data were compared to the curves predicted by the WB-PBPK model using estimated partition coefficients. Because there was a slight deviation of predicted to experimental plasma concentration profiles, the partition coefficients estimated from physico-chemistry for MDZ were compared to experimental partition coefficients, observed in rats and converted for use in humans, for MDZ [22]. With the carcass value being taken as that for bone, the correlation revealed that all predicted partition coefficients agreed with the experimental data within a factor of three with the exception of blood cells, bone, fat and lung, (Figure 8). For these tissues, the experimental partition coefficients were used in the MDZ simulations and the same predicted to experimental conversion factors were used for these tissues in the WB-PBPK models for 1OH-MDZ and 1OH-MDZ-Glu. An independent validation of the 1OH-MDZ-Glu distribution volume was not possible due to a lack of plasma concentration time data following 1OH-MDZ-Glu administration.

### Optimization of velocity-rate Constants for the hydroxylation of Midazolam

All relevant model parameters are now defined as described above with the exception of the biotransformation parameters. To generate velocity rate constants for the elimination of MDZ to 1OH-MDZ, individual plasma concentration time data for MDZ and its metabolite 1OH-MDZ following intravenous administration were gathered from Heizmann et al. [11]. Six normal healthy volunteers with reported body weight and age were enrolled in this study.

For each individual from the Heizmann et al. [11] study, WB-PBPK models were generated for MDZ and 1OH-MDZ. These two models were coupled and consequently contained four unknown parameters, 1OH_k_1_, 1OH_k_-1 _and 1OH_k_2_, which describe MDZ hydroxylation by CYP3A4/5 as in Eq. (1), and a first order elimination rate of OH-MDZ via an intrinsic clearance CL(1OH), which describes the glucuronidation of 1OH-MDZ. These four parameters were simultaneously optimized per individual, without constraints, in order to obtain the best fit of the simulated concentration time curves for MDZ and 1OH-MDZ in plasma to the respective experimental data. The objective function was the root mean squared error (RMSE) measured as the root of the squared difference between the predicted and observed plasma concentrations for both compounds. Objective function minimization was done using the *fminsearch *optimization routine of MatLab [Version 7 (R14), The MathWorks Inc., Natick, MA, USA].

### Optimization of velocity-rate constants for the Glucuronidation of 1-hydroxymidazolam

Plasma concentration time data for MDZ, its metabolite 1OH-MDZ and its conjugated metabolite 1OH-MDZ-Glu following intravenous bolus MDZ administration were gathered from Kharasch et al. [16]. Data from one typical representative was available and used here. The coupled WB-PBPK model for the Kharasch et al. [16] data contained seven unknown parameters, 1OH_k_1_, 1OH_k_-1 _and 1OH_k_2_, Glu_k_1_, Glu_k_-1 _and Glu_k_2_, and CL(Glu). These seven parameters were simultaneously optimized in order to obtain the best fit of simulated concentration time curves for MDZ, 1OH-MDZ and 1OH-MDZ-Glu to the corresponding experimental data. For the optimization of the elimination of MDZ to its metabolite 1OH-MDZ, the results of the previous optimization were used as constraints by setting the min and max constraints for 1OH_ k_1,-1,2 _as half of and two times the min and max values, respectively, as determined during the optimization of the six Heizmann et al. [11] individuals. The mean values of the optimization of the Heizmann et al. [11] data were used as initial parameters for 1OH_k_1,-1,2_. The optimization of the velocity-rate constants for the metabolization of 1OH-MDZ to its metabolite 1OH-MDZ-Glu were unconstrained, as was the first order elimination of 1OH-MDZ-Glu [CL(Glu)].

### Optimization of the velocity rate constants for the Yu et al (2004) study data in the Basal state

Individual plasma concentration time data for MDZ and the sum of its metabolites, 1OH-MDZ and 1OH-MDZ-Glu, in the basal state were taken from Yu et al. [4]. In this study, plasma concentrations of MDZ and 1OH-MDZ were determined (after addition of glucuronidase to the plasma samples, i. e. 1OH-MDZ concentrations represent the sum of 1OH-MDZ and 1OH-MDZ-Glu) for nineteen healthy adults having either the CYP3A5 *1/*1, *1/*3 or *3/*3 genotype. Individual values for height, body weight, sex, age and genotype were also provided [4].

A coupled WB-PBPK model was set up for each individual in the Yu et al. [4] study. Each model contained seven unknown parameters, 1OH_k_1_, 1OH_k_-1 _and 1OH_k_2_, Glu_k_1_, Glu_k_-1 _and Glu_k_2 _and CL(Glu). These seven parameters were again simultaneously optimized as described above in order to obtain the best fit of simulated concentration time curves for MDZ and the sum of 1OH-MDZ and 1OH-MDZ-Glu to the corresponding experimental data. The same constraints as above were used for the optimization of the elimination of MDZ to 1OH-MDZ. The initial values for optimization for Glu_ k_1,-1,2_are the results of the optimization of the Kharasch et al. data[16]. As Kharasch et al. [16] presented data from only one individual, there were no minima and maxima which could be used as constraints, therefore no constraints were used for the 1OH-MDZ-Glu parameters.

### Itraconazole CYP3A4 inhibition model

Itraconazole (ITZ) and its metabolites competitively inhibit CYP3A4 [13]. The major, and only significant, metabolite in vivo is hydroxyitraconazole (OH-ITZ) which is found at concentrations higher than ITZ in plasma [17].

First, a WB-PBPK model for ITZ was developed using the physicochemical properties as presented in Table 2. Elimination, representing CYP3A metabolism, was defined as a Michaelis-Menten process in the liver with a K_m _of 3.9 ηM [13] and a V_max _to fit the terminal phase of the plasma concentration profile from Barone et al. [17].

According to the study protocol in Yu et al. [4], the volunteers received a single oral dose of a commercial ITZ tablet, Sporanox^® ^(Janssen Pharmaceutica), in the fasted state over five days. Since the pharmacokinetics of ITZ and OH-ITZ were not assessed by Yu et al., the literature was searched for similar pharmacokinetic data. Barone et al. [17] provided plasma concentration time data for Sporanox^® ^tablets, but administered in the fed state. ITZ is a basic compound with a pKa of 3.7, and it is well known that administration of ITZ together with food increases the bioavailability due to a longer retention of the tablet in the stomach. Consequently, a modified dissolution profile had to be generated for the Sporanox^® ^tablet in the fasted state. Sporanox^® ^tablets are well dissolved in acidic solutions and, within 45 minutes, 50% are dissolved in an artificial stomach solution [40]. In contrast, in Fed State Simulating Intestinal Fluid (FeSSIF), Sporanox^® ^tablets reach a maximum fraction dissolved of 2% in 30 minutes [40]. In the WB-PBPK model, dissolution was set as 9, 35, 50% dissolved in 15, 30 and 45 minutes (the average length of time the tablets reside in the stomach in the fed state) to 52% in 75 minutes (2% being the maximum dissolution in the intestines). Further, ITZ is metabolized by CYP3A in the intestine. To account for gut wall metabolism, in each intracellular section of the gastrointestinal tract (duodenum, jejunum, ileum), a Michaelis-Menten process in the gut wall was assumed with a K_m _the same as that for hepatic metabolism (3.9 nM). A V_max _value for gut wall metabolism was fit to best represent the absorption phase of the Barone et al. [17] data. Only one V_max _required fitting since the proportions of CYP3A in each intestinal section are known (duodenum: jejunum: ileum = 9.5: 6.3: 1) [41]. The resulting single dose administration simulation was fit to experimental data from Barone et al. [17].

ITZ and OH-ITZ are primarily metabolized by CYP3A4. Since both have a high affinity for CYP3A4, saturation of the enzymes can lead to non-linear kinetics. Following multiple administrations, ITZ and OH-ITZ clearances are reduced and the concentrations in plasma of the two substances increase as well as the half-lives. In the Yu et al. [4] study, ITZ was administered once-daily for five days and on the fifth day given in conjunction with an IV midazolam bolus. To simulate the likely ITZ concentrations over 6 days, the time dependence of ITZ elimination was assessed using the data of Hardin et al. [18]. In this study, trough plasma concentrations were taken at days 2, 7, 11 and 13 during 200 mg once-daily administrations of ITZ in the fed-state. V_max _at time 0 was the same as that previously defined from the single dose study of Barone et al. [17]. The V_max _was reduced daily to fit the trough concentrations from Hardin et al. [18] (Figure 7).

The next step was to generate the fasted state ITZ and OH-ITZ liver unbound concentration time profiles that will be used to calculate the time specific CYP3A activity reduction (see next section). Since most literature data providing pharmacokinetic profiles of ITZ and OH-ITZ were those following ITZ administration in the fed state, from the multiple dose ITZ fed-state profiles (as described above), both the ITZ and OH-ITZ fasted-state profiles needed to be generated. Since the difference between the fed and fasted state plasma profiles are mainly due to differences in tablet dissolution, concentration differences over 24 hours are primarily the ratio of AUCs (AUC_fed _= 1.7*AUC_fasted_) [17]. This ratio remained the same throughout a 15 day BID administration regimen [17]. Therefore, the unbound concentrations in the liver intracellular space were taken from the WB-PBPK model for ITZ_fed _and divided by 1.7 to generate the relevant concentrations for ITZ_fasted_. For OH-ITZ_fasted_, the AUC ratio of ITZ_fed _to OH-ITZ_fasted _was used to generate concentrations where AUC_OH-ITZ,fasted _= 1.6* AUC_ITZ,fed _[17]. The relevant liver concentrations were therefore the ITZ_fed _unbound liver concentrations multiplied by 1.6 and divided by the ratio of unbound fractions (f_u,ITZ_/f_u,OH-ITZ _= 0.8). The unbound fraction of OH-ITZ is not available in the literature therefore the same ratio of parent to hydroxylated f_u_, as was determined for MDZ (Table 2), was used.

**Figure 9:**
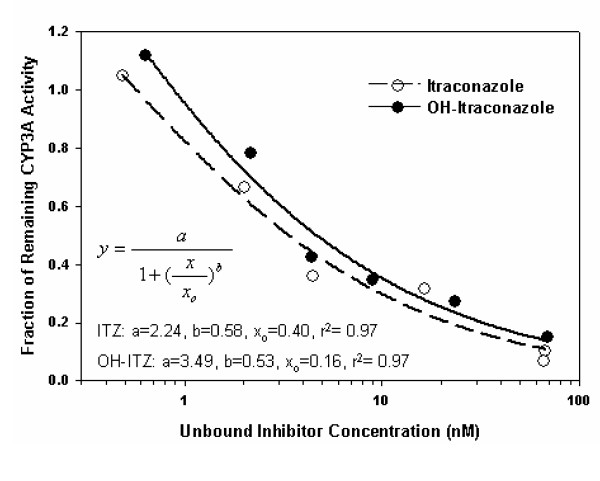
Fraction of remaining CYP3A activity in pooled human liver microsomes in relation to the unbound inhibitor concentrations of ITZ and OH-ITZ. Data points, as taken from Isoherranen et al. [13], were fit to a logistic, 3 parameter model and the equations were used to generate a percentage of CYP3A activity remaining, constrained between 0 and 1.

The effect of the unbound ITZ and OH-ITZ concentrations on CYP3A4 activity in vitro using MDZ hydroxylation as an endpoint has been assessed by Isoherranen et al. [13] and is presented in Figure 9. The unbound ITZ and OH-ITZ concentrations in the intracellular space of the liver, as obtained by the WB-PBPK model, were used in conjunction with Figure 9 to generate a remaining percentage of CYP3A activity. At each time step, a new CYP3A concentration ([CYP3A_i_]) was calculated using the following equation:

[CYP3A_i_] = [CYP3A]*ITZ_frac·act _*OH - ITZ_frac·act _    (6)

Here, ITZ_frac·act _and OH-ITZ_frac·act _are the fraction of the initial CYP3A activity based on ITZ and OH-ITZ concentrations from Figure 9 (constrained to values between 0 and 1).

### Prediction of MDZ and MDZ metabolite pharmacokinetic profiles following ITZ administration

**Figure 10:**
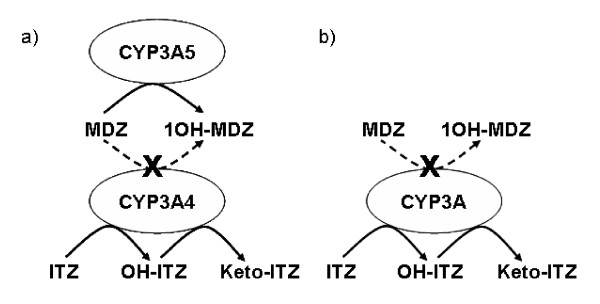
Schematic representation of the inhibition of the CYP3A4-catalyzed MDZ hydroxylation by ITZ and its major metabolite OH-ITZ (a) and the simplified model used in this study (b).

Coupling of the previously established MDZ and ITZ models (a total of 5 WB-PBPK models) finally facilitates the prediction of the effect of ITZ and OH-ITZ CYP3A inhibition in the pharmacokinetics of MDZ and its metabolites. The coupling was done such that the clearance of MDZ was affected by the changing concentrations of free CYP3A [E_o_] as defined from the ITZ inhibition model (Figure 10). WB-PBPK Simulations were performed for each of the individuals in the Yu et al. [4] study. All velocity-rate constants as well as CL(Glu) were fixed to the optimized values determined previously in the simulations for the basal state for each individual. Results were compared to the MDZ and 1OH-MDZ + 1OH-MDZ-Glu plasma concentration profiles. In summary, the assumptions of this combined model were:

1/ *MDZ and metabolite elimination kinetics can be adequately described using Michaelis-Menten kinetics*.

2/ The *1'-hydroxylation step is the only significant route of elimination for midazolam from the body. *4OH-MDZ and 1,4-di-OH-MDZ plasma pharmacokinetic profiles are not published since they are not found in significant amounts in plasma [15]. Furthermore, as the percentage of administered drug excreted in the urine as 4OH-MDZ and 1,4-di-OH-MDZ (or their glucuronides) is less than 4%, suggesting that these MDZ elimination pathways are not significant.

3/ *The physico-chemical properties of the compounds were adequate to describe the compound's distribution volume. *This was independently evaluated for MDZ, 1OH-MDZ and ITZ using experimental plasma concentration time data following IV administration and using some MDZ experimental partition coefficients.

4/ *The CYP3A complex was adequate to describe all (CYP3A4 and CYP3A5) CYP3A metabolic processes*.

5/ *ITZ and OH-ITZ affect the total active CYP3A concentration in the liver*.

6/ *ITZ and OH-ITZ unbound liver intracellular concentrations were adequate representations of actual concentrations in the volunteers from the Yu et al*. [4]*study*.

## Abbreviations

**AUC **Area under the curve

**CL(Glu) **Intrinsic clearance of 1'hydroxymidazolam glucuronide

**CL(1OH) **Intrinsic clearance of 1'hydroxymidazolam

**CV% **Coefficient of variation

**CYP **Cytochrome P450

**E**_**o **_Enzyme concentration

**ES **Enzyme-substrate complex

**ITZ **Itraconazole

**IV **Intravenous

**Km **Michaelis-Menten constant

**MDZ **Midazolam

**OH-ITZ **Hydroxyitraconazole

**1OH-MDZ **1' Hydroxymidazolam

**1OH-MDZ-Glu **1' Hydroxymidazolam glucuronide

**P **Product

**S, [S] **Substrate, substrate concentration

**UGT **UDP-glucuronosyltransferase

**Vmax **Maximum velocity of an enzymatic reaction

**WB-PBPK **Whole body physiologically-based pharmacokinetic

## Competing interests

MV, MS, CN, SW, and ANE are employees of Bayer Technology Services GmbH.

## Authors' contributions

MV designed and performed the basal state MDZ simulations, MS conceptualized and designed the computational system to perform the simulations, CN and SW provided valuable inputs into the conceptual design of the study and helped to draft the manuscript, IJ provided experimental data and data interpretation for the study, and ANE conceived of the study and designed and performed the inhibited state MDZ-ITZ simulations. All authors read and approved the final manuscript.
